# The Critically Endangered Bolivian Endemic *Hieronymiella bedelarii* (Amaryllidaceae): First Alkaloid Profile, Cholinesterase Inhibition, and Ex Situ Conservation Insights

**DOI:** 10.3390/plants15203125

**Published:** 2026-10-13

**Authors:** Olimpia Llalla-Córdova, Javier E. Ortiz, Luciana R. Tallini, Victoria Parera, Laura Torras-Claveria, Lorena C. Luna, Gabriela E. Feresin

**Affiliations:** 1 Consejo Nacional de Investigaciones Científicas y Técnicas (CONICET), CCT CONICET San Juan, Av. Libertador General San Martín 1109 (O), San Juan 5400, Argentina; olimpiallcr@gmail.com (O.L.-C.);; 2 Instituto de Biotecnología, Facultad de Ingeniería, Universidad Nacional de San Juan, Av. Libertador General San Martín 1109 (O), San Juan 5400, Argentina; 3 Escuela Profesional de Ingeniería Agroindustrial, Universidad Nacional de Moquegua, Prolongación Calle Ancash s/n, Moquegua 18001, Peru; 4 Departament de Biologia, Sanitat i Medi Ambient, Facultat de Farmàcia i Ciències de l’Alimentació, Universitat de Barcelona, Av. Joan XXIII 27-31, 08028 Barcelona, Spain

**Keywords:** Eustephieae, candimine, GC–MS, UPLC–MS/MS, acetylcholinesterase, bulbil establishment, *Hieronymiella*, Bolivian Andes

## Abstract

*Hieronymiella bedelarii* is a Bolivian endemic species known from a single locality in Torotoro National Park and currently assessed as Critically Endangered on the IUCN Red List. Its alkaloid composition and cholinesterase-inhibitory activity have not previously been investigated. The GC–MS analysis identified eighteen alkaloids representing six structural types, with lycorine-, pretazettine- and homolycorine-type alkaloids being the most abundant groups. Bulbs of *H. bedelarii* yielded an alkaloid-enriched extract (0.80% *w*/*w*). Eight of the identified alkaloids are reported here for the first time in *Hieronymiella*, including candimine. Complementary UPLC–MS/MS analysis detected pretazettine and the artifact tazettine in the alkaloid-enriched extract. This extract showed stronger inhibition of acetylcholinesterase than butyrylcholinesterase, with IC_50_ values of 10.18 and 193.19 µg·mL^−1^, respectively. In a preliminary cultivation trial, 11 of 20 bulbils (55%) established and developed roots and leaves after 12 months under nursery conditions.

## 1. Introduction

Amaryllidaceae alkaloids constitute a distinctive group of specialized metabolites derived from isoquinoline, which are characteristic of the Amaryllidoideae subfamily. Most Amaryllidaceae alkaloids derive from the common precursor 4′-*O*-methylnorbelladine, formed through the norbelladine pathway from L-phenylalanine and L-tyrosine, and undergo oxidative phenol coupling that gives rise to remarkable structural diversity, currently classified into 42 skeleton types [1]. These alkaloids exhibit a broad range of biological activities, including anticholinesterase, antiprotozoal, anti-inflammatory, antioxidant, and cytotoxic effects [2]. Among these activities, cholinesterase inhibition has received particular attention because galanthamine, an Amaryllidaceae alkaloid and reversible acetylcholinesterase inhibitor, is used clinically for the symptomatic treatment of mild to moderate Alzheimer’s disease [3].

Gas chromatography–mass spectrometry (GC–MS) is the standard technique for the analysis of Amaryllidaceae alkaloids [1], but some compounds are difficult to identify or quantify reliably by this approach. Homolycorine-type alkaloids with a C3–C4 double bond, for example, give low-intensity molecular and diagnostic ions under electron-impact ionisation, which can lead to ambiguous identifications in complex plant extracts [4]. Artifacts formed during extraction or analysis can also alter the profile. Pretazettine, for instance, converts to tazettine under the basic conditions of alkaloid extraction and during GC–MS analysis, so the two cannot be distinguished by this technique [5,6]. Electrospray ionisation tandem mass spectrometry (ESI–MS/MS) provides complementary fragmentation data that can assist in such cases [6].

The tribe Eustephieae Hutch. (Amaryllidaceae, subfamily Amaryllidoideae), comprising the Andean genera *Eustephia* Cav., *Hieronymiella* Pax, *Pyrolirion* Herb., and *Chlidanthus* Herb., constitutes a small clade within the Andean Amaryllidoideae [7]. Phytochemical studies of *Hieronymiella* have revealed diverse Amaryllidaceae alkaloid profiles and variation among species and collection sites. In five species collected at different localities in Argentina, GC–MS analysis showed differences in alkaloid composition among samples, including chemical differences within the same species, and their alkaloid extracts inhibited acetylcholinesterase [8]. More recently, analysis of bulbs and leaves of *H. peruviana* also revealed differences in alkaloid composition and relative abundance between plant organs, together with cholinesterase inhibitory activity [9]. However, these studies cover only the southern and northern portions of the genus distribution, whereas the Bolivian inter-Andean valleys remain phytochemically unexplored.

The Central Andes, including the Bolivian inter-Andean valleys, host a highly diverse flora with remarkable levels of endemism, yet remain particularly vulnerable to climate change. Current projections suggest significant shifts in the distribution of endemic plant species across the southern Central Andes, underscoring the heightened vulnerability of geographically restricted taxa [10,11]. These concerns are particularly relevant for narrowly distributed species, for which biological and ecological information can be important for conservation planning.

*Hieronymiella bedelarii* R. Lara & H. Huaylla is a Bolivian endemic species described from Cerro Llamachaki in the municipality of Torotoro, Department of Potosí, Bolivia [12]. The species is currently assessed as Critically Endangered (CR) on the IUCN Red List, with a single known locality and an estimated area of occupancy of 4 km^2^, and no ethnomedicinal uses have been documented [13]. No current ex situ collection of *H. bedelarii* was identified in Botanic Gardens Conservation International (BGCI) PlantSearch [14] or in the publicly available records of the Bolivian botanical gardens consulted. Based on its floral and vegetative characters, *H. bedelarii* is considered closely related to *H. peruviana* [15], and its study addresses the phytochemical gap in the central portion of the genus distribution.

Bulbous geophytes can reproduce sexually through seed production or vegetatively through structures such as bulbils, which arise by natural division from the parent bulb [16]. In some Amaryllidoideae, bulbils originate from axillary buds formed at the junction between the basal plate and the innermost bulb scales and gradually develop into independent propagules [17]. Under nursery or greenhouse conditions, the establishment and subsequent development of bulb-derived material can be influenced by substrate conditions [18]. Evaluating the establishment of bulbils under cultivation can provide useful information for maintaining living plants of species with restricted distributions, such as *H. bedelarii*.

The aim of this study was to characterize the bulb alkaloid profile of *H. bedelarii* using GC–MS, complemented by UPLC–MS/MS analysis, and to evaluate the inhibitory activity of the alkaloid-enriched extract against acetylcholinesterase and butyrylcholinesterase enzymes. In addition, a preliminary establishment trial was conducted using bulbils.

## 2. Results and Discussion

### 2.1. Field Observations and Site Conditions

**Figure 1:**
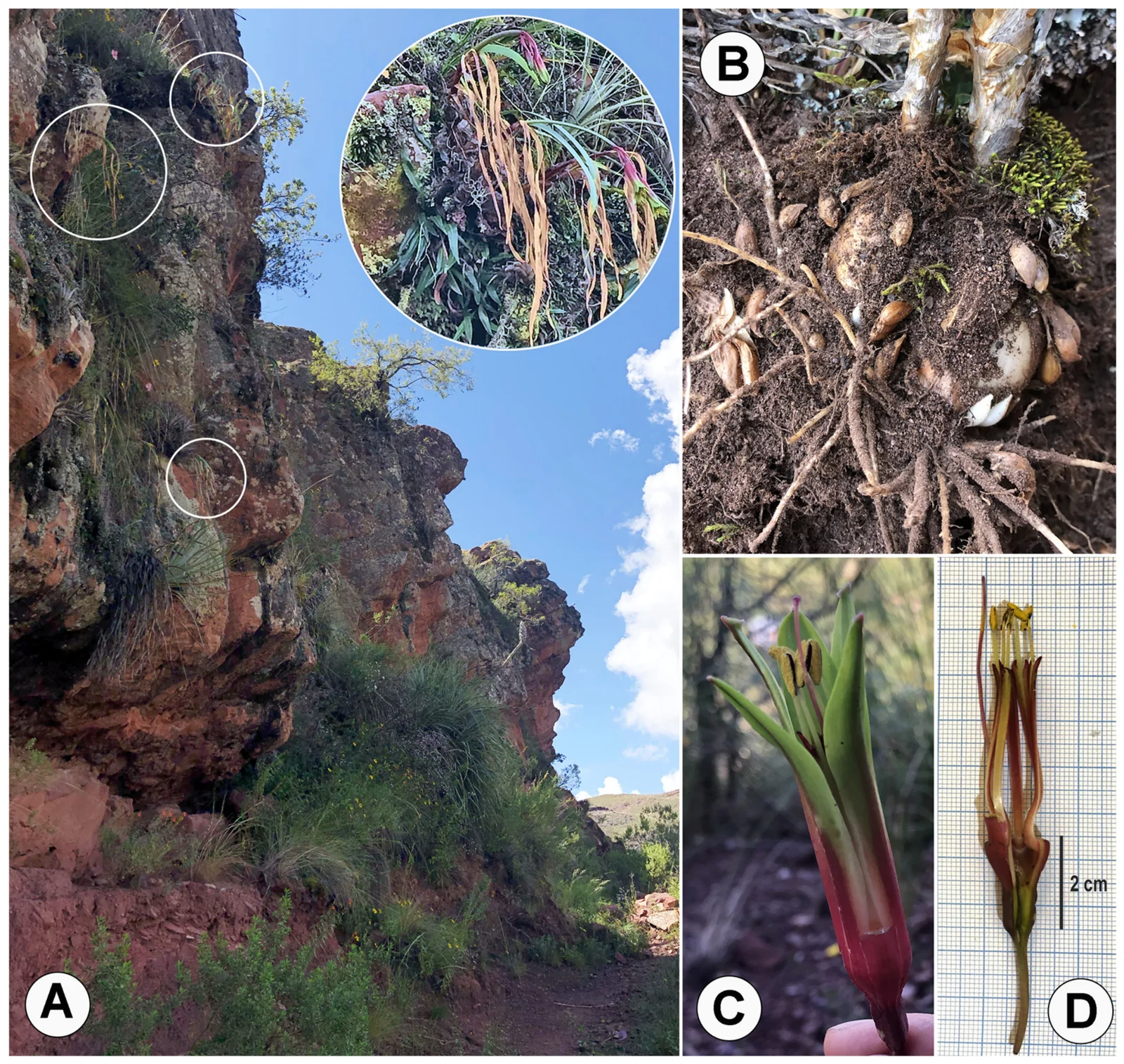
*Hieronymiella bedelarii* at its type locality. (**A**) Natural habitat on steep rocky slopes (circles). (**B**) Bulb with bulbils. (**C**) Flower, lateral view. (**D**) Staminal filaments. (Photographs by O. Llalla-Cordova).

*Hieronymiella bedelarii* grows on steep, poorly accessible rocky slopes at Cerro Llamachaki, Torotoro, Potosí, Bolivia, characterized by shaded, moist crevices and ledges (Figure 1A). Additional populations may occur in comparable rocky habitats elsewhere in the region. Adult and juvenile plants were observed, together with numerous bulbils arising from the basal region of adult bulbs (Figure 1B), a feature not documented in the original description of the species [12]. Detached bulbils were ovoid-fusiform, 1.0–1.5 cm high and 0.5–0.7 cm wide. Shaded, moist rock crevices were observed among the rocky slopes. No vernacular name was reported by local people consulted at the collection site.

ERA5-Land estimates for 2021–2024 [19], extracted from the nearest grid cell to the collection locality, approximate the site’s microclimate and indicated a strongly seasonal precipitation regime, with 646 mm accumulated on average between December and April compared with 43 mm between May and August, while annual precipitation ranged from 422.6 to 1241.1 mm; monthly mean temperatures ranged from 12.4 to 19.3 °C (Table S1). These estimates provide climatic context for the phenological condition observed in April 2024, when plants exhibited advanced leaf senescence, few remaining flowers, and no mature fruits. This condition corresponds to the hysteranthous phenology described for the species, which develops and flowers with the onset of the rainy season and may continue into March or April [12]. The morphology of the staminal filaments (Figure 1 C,D), particularly their appendages, provides diagnostic characters distinguishing *H. bedelarii* from the closely related *H. peruviana* [15].

### 2.2. Alkaloid Profile

Bulbs of *Hieronymiella bedelarii* yielded 0.80% (*w*/*w*) of alkaloid-enriched extract. GC–MS identified eighteen alkaloids across six structural types (Table 1). Lycorine-type alkaloids were the most abundant structural group (33.69 µg Gal/100 mg DW), followed by pretazettine- (25.04 µg Gal/100 mg DW) and homolycorine-type alkaloids (20.75 µg Gal/100 mg DW). Identified alkaloids totalled 100.35 µg Gal/100 mg DW, with a further 22.10 µg Gal/100 mg DW from six unidentified peaks. These values are semi-quantitative estimates expressed as galanthamine equivalents, and should not be interpreted as absolute concentrations.

The alkaloid profile of *H. bedelarii* differed from those previously reported for other *Hieronymiella* species, both in overall composition and representation of structural types. Anhydrolycorine and lycorine were consistently detected across the previously reported profiles within this genus, establishing the lycorine-type alkaloids as the most widely represented structural group, whereas other skeleton types appeared more restricted in distribution [8,9]. *H. bedelarii* shares homolycorine-type alkaloids with *H. peruviana* [9] and pretazettine- and crinine/haemanthamine-type alkaloids *H. caletensis* and *H. aurea*, respectively [8]. Notably, eight of the eighteen alkaloids identified are reported here for the first time in the genus *Hieronymiella*: assoanine, galanthine, incartine, 1-*O*-acetyllycorine, candimine, epimacronine, *O*-methyltazettine, and galanthindole.

**Figure 2:**
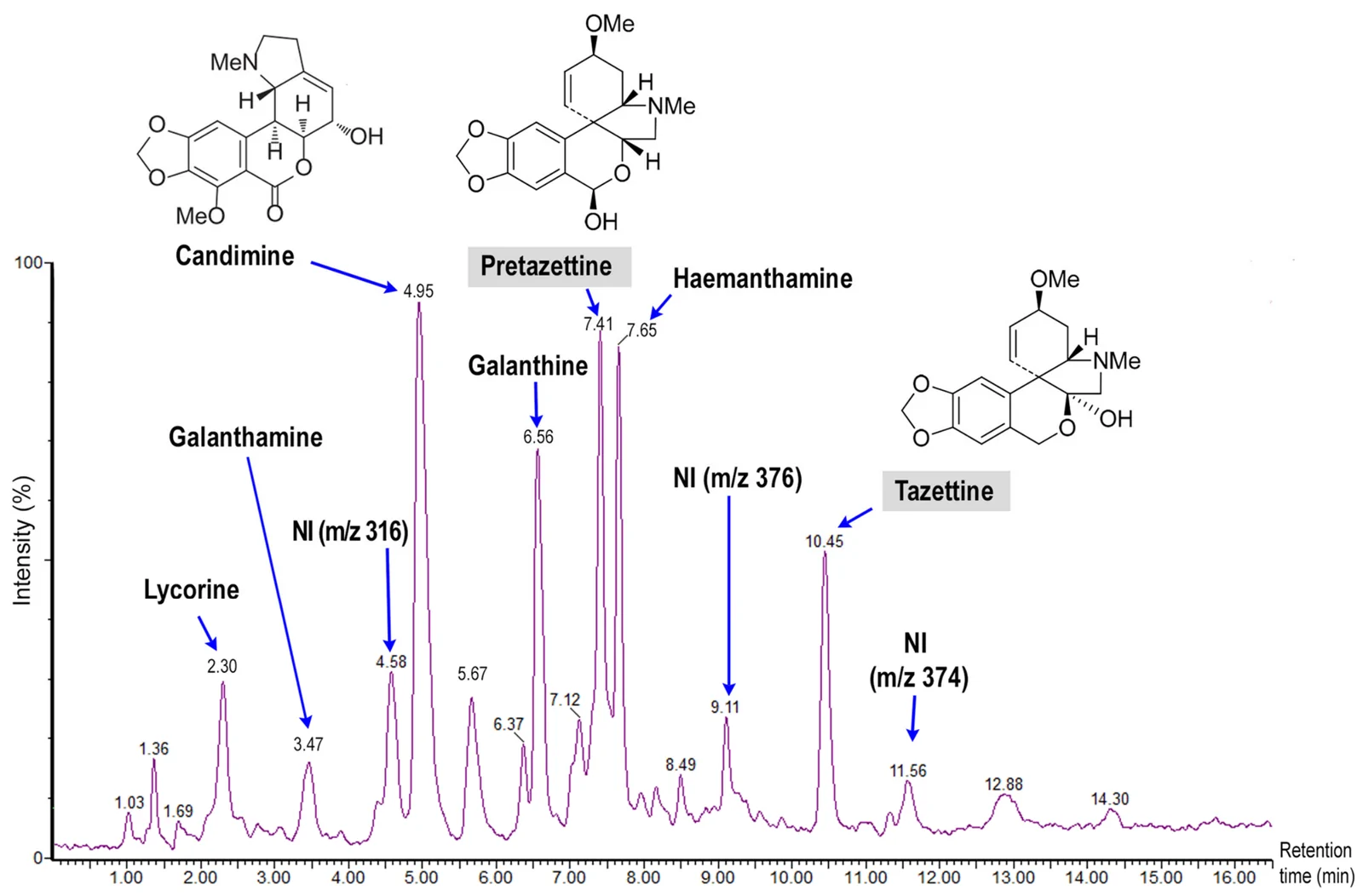
UPLC–MS/MS chromatogram (ESI^+^, full-scan MS/MS, CE 30 eV) of the alkaloid-enriched extract of *Hieronymiella bedelarii* bulbs, showing the main identified alkaloids and the separation of pretazettine (RT 7.41 min) and tazettine (RT 10.45 min).

Tazettine was the most abundant individual component identified by GC–MS (18.41 µg Gal/100 mg DW). This alkaloid is widely regarded as an artifact of pretazettine, which is essentially undetectable by GC–MS because it converts to tazettine both during alkaline extraction and within the chromatographic system itself [6]. As a complement to GC–MS, UPLC–MS/MS analysis resolved both compounds, which share the same [M+H]^+^ ion (*m*/*z* 332), but differ in MS/MS fragmentation and retention time, in agreement with ESI-QToF-MS/MS data reported for both compounds [5] (Figure 2; Table S2). Pretazettine persisted in the alkaloid-enriched extract alongside tazettine. Three compounds were detected whose fragmentation patterns were consistent with a tentative assignment to the homolycorine-type alkaloids, with molecular masses of 316, 376, and 374 Da and retention times of 4.58, 9.11, and 11.56 min, respectively. In addition, several signals could not be confidently assigned to any specific Amaryllidaceae alkaloid type.

Several of the identified alkaloids have previously reported biological activities. Pretazettine has shown cytotoxic activity against several tumor cell lines, whereas its rearrangement product tazettine has generally shown weaker or no activity in comparable models [25]. Another alkaloid identified in the bulbs of *H. bedelarii*, candimine, has previously shown in vitro trypanocidal activity against *Trypanosoma cruzi* [26]. Its occurrence in *H. bedelarii* adds an alkaloid with previously reported biological activity to the known chemical repertoire of the genus.

### 2.3. Cholinesterase Inhibitory Activity

The alkaloid-enriched extract of *Hieronymiella bedelarii* showed a stronger inhibitory effect on AChE than on BuChE, with a selectivity index (SI) of 19.0 (Table 2). The AChE inhibitory activity falls within the range reported for *Hieronymiella* species collected in Argentina (1.84–15.40 µg·mL^−1^) [8], and is comparable to that of *H. peruviana* (AChE IC_50_ = 8.60 µg·mL^−1^) [9]. In contrast, the BuChE activity of *H. bedelarii* is close to the limit considered inactive (IC_50_ > 200 µg·mL^−1^).

Activity varies among the alkaloids present. Within the lycorine type, Elgorashi et al. (2004) [27] reported AChE inhibitory activity for 1-*O*-acetyllycorine (IC_50_ = 0.96 µM), whereas lycorine showed weak activity (IC_50_ = 213 µM). Ortiz et al. [8] demonstrated that tazettine, despite being the most abundant individual alkaloid, exhibited no inhibitory activity against either enzyme when tested as an isolated compound (>200 µg·mL^−1^). Galanthamine accounted for approximately 6.5% of the total alkaloid content estimated by semi-quantitative GC–MS (Table 1), and may contribute, at least in part, to the AChE inhibitory activity of the alkaloid-enriched extract. Other identified and unidentified alkaloids may also contribute to the observed activity.

### 2.4. Preliminary Bulbil Establishment Under Cultivation

**Figure 3:**
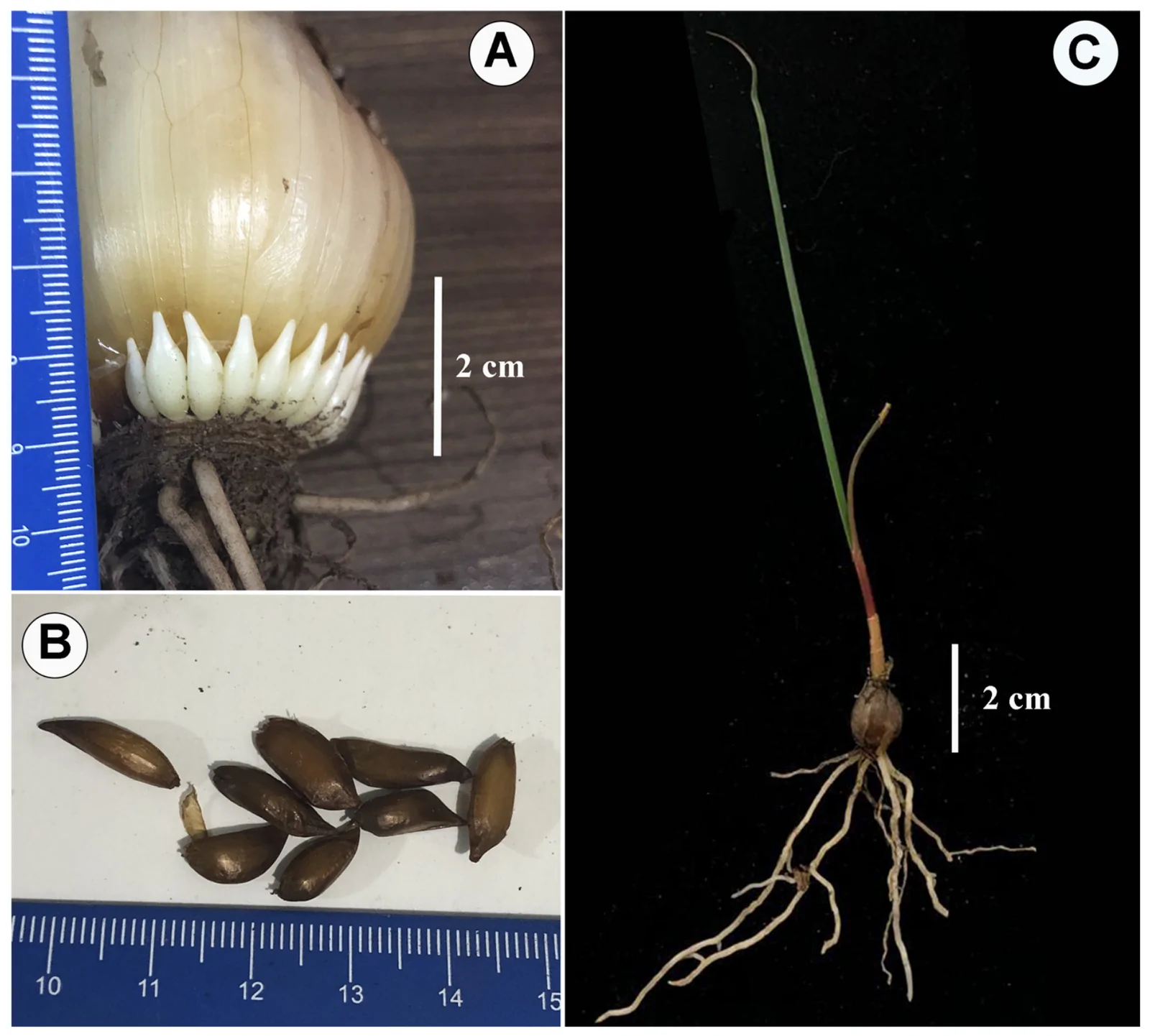
(**A**) Adult bulb bearing bulbils. (**B**) Loose bulbils. (**C**) Representative plant after 12 months under cultivation. (Photographs by O. Llalla-Cordova).

Bulbils are vegetative propagules capable of developing into new plants [28]. In *Hieronymiella bedelarii*, bulbils were found loose in the surrounding substrate (Figure 3B) and were used in this assessment. After 12 months, establishment was assessed. Eleven bulbils (55%) developed adventitious roots and leaves (Figure 3C). The established bulbils measured 1.15 ± 0.12 cm in height and 0.95 ± 0.07 cm in width, with 6.9 ± 1.6 roots per bulbil, a mean root length of 5.0 ± 1.3 cm and a leaf length of 11.1 ± 0.9 cm. The remaining nine bulbils were found with collapsed or disintegrated tissue, suggesting loss rather than dormancy.

Establishment in bulbous plants depends partly on substrate conditions, particularly the balance between aeration and moisture [18]. Cold storage duration, substrate type, temperature and watering regime remain to be evaluated for this species. The relative contribution of bulbils and seeds to recruitment in the wild population, where juvenile plants were observed, also remains unknown. The plants established here, as well as less destructive samples such as leaves or bulb scales, may provide material for further chemical and biological studies without additional collection from the wild.

## 3. Materials and Methods

### 3.1. Plant Material

Bulbs of *Hieronymiella bedelarii* were collected in April 2024 at Cerro Llamachaki, Torotoro, Potosí Department, Bolivia. At the time of collection, the leaves were in advanced senescence and few flowers were observed. Six bulbs were collected for chemical and bioactivity analyses. The plant material was identified by Dr Hibert Huaylla. Herbarium vouchers were deposited at LIL, MOQ, and LPB; voucher numbers LIL 618690 and MOQ 2043 are provided. Collection was authorized under permit MMAYA-VMABCCDF-DGBAP-UGOE-0397-CAR/24 issued by the Ministry of Environment and Water of Bolivia.

### 3.2. Preparation of the Alkaloid-Enriched Extract

Prior to extraction, the basal discs with their roots were separated and kept in pots. The remaining tissue from all six bulbs was pooled into a single composite sample to account for biological variability and obtain a representative chemical profile of the bulbs. This material was then cut into pieces, dried at 40 °C to constant weight, powdered, and macerated twice in methanol, using 50 mL of methanol per 10 g of dry material (72h each, in the dark, with occasional stirring). The solvent was evaporated under reduced pressure using a rotary evaporator (Heidolph Instruments GmbH & Co. KG, Schwabach, Germany); the residue was acidified to pH 3.5–4 with 2% H_2_SO_4_ and washed three times with ethyl ether to remove neutral compounds. The aqueous phase was alkalinised to pH 9–10 with 20% NaOH and extracted three times with ethyl acetate. The organic extracts were dried over anhydrous Na_2_SO_4_, filtered, and concentrated to dryness in vacuo to yield the alkaloid-enriched extract. Yields were calculated as percentages of the dry starting material. Methanol, H_2_SO_4_, and ethyl ether were purchased from Merck Química Argentina (Buenos Aires, Argentina).

### 3.3. GC–MS Conditions and Alkaloid Profile Identification

The alkaloid-enriched extract was dissolved in 1 mL of methanol containing 25 µg/mL of codeine (used as internal standard), and an aliquot of 1 µL was injected. Analysis was performed on an Agilent 6890N coupled to a 5975 mass detector (Agilent Technologies, Santa Clara, CA, USA) in electron impact mode (70 eV). Separation was performed on a DB-5 MS column (30 m × 0.25 mm × 0.25 µm) with temperatures ramped from 100–180 °C at 15 °C/min (1 min hold), then 180–300 °C at 5 °C/min (1 min hold). Injector: 280 °C; helium flow: 0.8 mL/min; split ratio 1:20. Data were processed with AMDIS 2.73, and the alkaloids were identified by comparing their EI mass spectra and Kovats retention indices with reference data from previously isolated and structurally characterized Amaryllidaceae alkaloids (by 1D and 2D NMR and HRMS analyses) in the alkaloid library of the Natural Products Research Group, University of Barcelona, together with the NIST 20 database and published literature. The Kovats retention indices were calculated using an n-alkane calibration mixture (C9–C36, Restek, Bellefonte, PA, USA, cat. no. 31614). Semi-quantitative results are expressed as µg galanthamine equivalent per 100 mg dry weight. Galanthamine, available as an authentic standard, shows stable behavior under GC–MS conditions [29], and its use as the reference unit allows direct comparison with the wider body of GC–MS-based Amaryllidaceae alkaloid literature.

### 3.4. UPLC-MS/MS Analysis

UPLC–MS/MS was used as a qualitative complement to GC–MS analysis of the alkaloid-enriched extracts. Chromatographic separation and data acquisition were performed on a Waters ACQUITY H-Class system coupled to a XEVO TQ-S micro triple quadrupole mass spectrometer (Waters Corp., Milford, MA, USA), with data processed using MassLynx V4.2 (Waters). The system operated in positive electrospray ionisation mode (ESI^+^), using an ACQUITY BEH C18 column (1.7 μm, 2.1 mm × 100 mm) at 35 °C. The mobile phase consisted of (A) 0.1% formic acid in water, (B) 0.1% formic acid in acetonitrile, and (C) methanol, at a flow rate of 0.2 mL min^−1^, with a gradient from 95% A–5% B (0–2 min) to 85% A–15% B (5 min), 80:10:10 A:B:C (10–14 min), and 60:20:20 A:B:C (17 min), returning to 95% A–5% B (18–20 min). Alkaloid-enriched extracts were dissolved in methanol–water (50:50, *v*/*v*) at 100 ppm, filtered (0.22 μm), and 10 μL were injected. Capillary voltage was 2 kV, cone voltage 43 V, and collision energy 30 eV (*m*/*z* range 50–1000). Alkaloid signals were annotated by comparison of precursor ions and MS/MS fragmentation patterns with an in-house library of previously isolated and structurally characterised Amaryllidaceae alkaloids. Signals lacking sufficient diagnostic evidence were reported as tentative or unidentified.

### 3.5. Evaluation of Cholinesterase Inhibition

AChE (from *Electrophorus electricus*) and BuChE (from equine serum), together with their substrates acetylthiocholine and butyrylthiocholine iodide, and galanthamine hydrobromide (purity ≥95% by HPLC; product No. 345670), were obtained from Sigma-Aldrich (St. Louis, MO, USA). Galanthamine was used as the reference inhibitor because of its established acetylcholinesterase-inhibitory activity. Inhibitory activity was measured using the spectrophotometric method described previously, with some modifications [30,31]. In each well of a 96-well microplate, 50 µL of AChE or BuChE enzyme solution (0.25 U·mL^−1^) and 50 µL of the alkaloid-enriched extract dissolved in phosphate-buffered saline (PBS; 8 mM K_2_HPO_4_, 2.3 mM NaH_2_PO_4_, 0.15 M NaCl, pH 7.5) were added and incubated at room temperature for 30 min. Subsequently, 100 µL of substrate solution containing DTNB and acetylthiocholine iodide (0.6 mM) or butyrylthiocholine iodide (0.24 mM), prepared in a saline solution with Na_2_HPO_4_ (pH 7.5), were added, and absorbance was recorded at 405 nm 5 min after the reaction began using a Multiskan FC microplate reader (Thermo Scientific, Waltham, MA, USA). The alkaloid-enriched extract was first screened at five concentrations (1–200 µg·mL^−1^) against both enzymes. For AChE, the IC_50_ was determined in a refined range of five concentrations (9–40 µg·mL^−1^); for BuChE, the IC_50_ was estimated from the initial screening range.

IC_50_ values were estimated by nonlinear regression using a four-parameter logistic model in RStudio (version 2026.07.1; Posit Software, PBC). Galanthamine and PBS served as positive and negative controls, respectively. The assays were performed in three independent experiments, each performed in triplicate.

### 3.6. Preliminary Evaluation of Bulbil Establishment Under Cultivation

Twenty detached bulbils, collected loose from the substrate surrounding adult bulbs, were stored at 5 °C for six months. They were then transferred to a single tray with individual cells (4.9 cm diameter, 9 cm height) filled with perlite:peat:sand (5:3:2, *v*/*v*/*v*) and maintained under nursery conditions, with temperatures of approximately 24 °C during the day and 12 °C at night, as recorded with a temperature logger (Elitech RC-4HC, Elitech, Xuzhou, China). Supplementary lighting was provided using a GrowTech 400 W LED grow light under a 12 h light/12 h dark photoperiod. Establishment and bulb dimensions were recorded after 12 months. Watering was reduced during the dry season (from May to August), identified from monthly ERA5-Land data for 2021–2024 [19], extracted for the collection coordinates, as direct meteorological measurements were not available at the site.

## 4. Conclusions

This study provides the first phytochemical characterization and cholinesterase inhibition assessment of *Hieronymiella bedelarii*, extending the known chemical diversity of the genus through eight alkaloids not previously reported, including candimine. UPLC–MS/MS complemented the GC–MS profile by resolving pretazettine from tazettine, an artifact derived from it. The stronger inhibition of AChE relative to BuChE is consistent with the cholinesterase-inhibitory activity reported for other *Hieronymiella* species. Several GC–MS signals remained unidentified and represent candidates for future isolation. The preliminary bulbil trial provided initial evidence of establishment under nursery conditions, although further evaluation is necessary to characterize establishment and subsequent growth. The plants established in this study may serve as a sustainable source of material for future work on this species, reducing the need for further collection in the wild.

## Figures and Tables

**Table 1:** Alkaloid profile of *H. bedelarii* determined by GC–MS. Values are expressed as µg of galanthamine equivalents (Gal) per 100 mg dry weight (DW).

N°	Alkaloid	[M]^+^	Base Ion	RI	Content (µg Gal/100 mg DW)	Reference
	**I. Lycorine-type (total)**				**33.69**	
1	Anhydrolycorine	251	250	2496.2	3.16	[20]
2	Assoanine	267	266	2566.1	3.08	[20]
3	Galanthine	317	242	2691.3	11.97	[20]
4	Sternbergine	331	228	2695.9	2.94	[20]
5	1-*O*-Acetyllycorine	329	226	2703.9	2.89	[21]
6	Lycorine	287	226	2735.9	1.70	[20]
7	Incartine	333	332	2736.8	7.95	[20]
	**II. Pretazettine-type (total)**				**25.04**	
8	*O*-Methyltazettine	345	261	2593.4	3.63	[22]
9	Pretazettine/Tazettine *	331	247	2635.8	18.41	[6]
10	Epimacronine	329	245	2798.7	3.00	[6]
	**III. Homolycorine-type (total)**				**20.75**	
11	*O*-Methyllycorenine	331	109	2475.4	2.93	[6]
12	Homolycorine	315	109	2791.9	3.01	[4]
13	2-Hydroxyhomolycorine	331	125	2938.3	6.06	[4]
14	Candimine	345	125	3038.9	8.75	[4]
	**IV. Galanthamine-type (total)**				**11.49**	
15	Galanthamine	287	286	2405.9	7.95	[23]
16	Narwedine	285	284	2469.3	3.54	[23]
	**V. Crinine/Haemanthamine-type (total)**				**5.70**	
17	Haemanthamine/Crinamine	301	272	2622.4	5.70	[24]
	**VI. Galanthindole-type (total)**				**3.68**	
18	Galanthindole	281	281	2489.3	3.68	[6]
	**Not identified (total)**				**22.10**	
19	NI-1	299	238	2448.7	4.72	
20	NI-2 (Lycorine-type)	315	228	2504.5	3.07	
21	NI-3	285	238	2519.5	2.90	
22	NI-4	375	374	2808.8	3.35	
23	NI-5	373	372	2834.3	3.54	
24	NI-6 (Homolycorine-type)	nd	125	2868.3	4.52	

* Detected as tazettine by GC–MS; NI, not identified; nd, not determined; RI, retention index; [M]^+^, molecular ion. Identified and NI components total 100.35 and 22.10 µg Gal/100 mg DW, respectively (overall, 122.45). Bold type indicates structural-type headings and the corresponding group subtotal values.

**Table 2:** Inhibitory activity of the alkaloid-enriched extract against acetylcholinesterase (AChE) and butyrylcholinesterase (BuChE).

Extract	AChE IC_50_	BuChE IC_50_	SI
*H. bedelarii* (Alkaloid-enriched extract)	10.18 ± 0.75 µg·mL^−1^	193.19 ± 4.25 µg·mL^−1^	19.0
Galanthamine (Positive control)	1.34 ± 0.02 µM	18.61 ± 0.39 µM	13.7

Data are expressed as mean ± standard deviation of three independent experiments (*n* = 3), each performed in triplicate. SI = Selectivity Index (BuChE/AChE).

## Data Availability

Data are contained within the article and Supplementary Materials.

## Supplementary Materials

The following supporting information can be downloaded at https://www.mdpi.com/article/10.3390/plants15203125/s1.
