# Supplementary material for: The Critically Endangered Bolivian Endemic Hieronymiella bedelarii (Amaryllidaceae): First Alkaloid Profile, Cholinesterase Inhibition, and Ex Situ Conservation Insights
Source: Plants (Basel). 2026 Oct 13;15(20):3125. doi: 10.3390/plants15203125 (PMC13709222; doi:10.3390/plants15203125)
Supplement: Supplementary file 1 [file plants-15-03125-s001.zip › plants-4567411-supplementary.pdf]

*Supplementary materials*

# The critically endangered Bolivian endemic *Hieronymiella bedelarii* (Amaryllidaceae): first alkaloid profile, cholinesterase inhibition, and *ex situ* conservation insights


**Olimpia Llalla-Córdova [1,2,3], Javier E. Ortiz [2], Luciana R. Tallini [4], Victoria Parera [1,2], Laura Torras-Claveria [4], Lorena C. Luna [2] and Gabriela E. Feresin [1,2*]**

1 Consejo Nacional de Investigaciones Científicas y Técnicas (CONICET), CCT CONICET San Juan, Av. Libertador General San Martín 1109 (O), San Juan CP5400, Argentina; olimpiallcr@gmail.com (O.L.-C.)

2 Instituto de Biotecnología, Facultad de Ingeniería, Universidad Nacional de San Juan, Av. Libertador General San Martín 1109 (O), San Juan CP5400, Argentina

3 Escuela Profesional de Ingeniería Agroindustrial, Universidad Nacional de Moquegua, Prolongación Calle Ancash s/n, Moquegua 18001, Peru

4 Departament de Biologia, Sanitat i Medi Ambient, Facultat de Farmàcia i Ciències de l'Alimentació, Universitat de Barcelona, Av. Joan XXIII 27-31, 08028 Barcelona, Spain

* Correspondence Author: Gabriela Feresin, email: gferesin@unsj.edu.ar; Tel.: +54-264-421-1700 (ext. 410/294); Fax: +54-264-420-0289

**Table S1.** Monthly precipitation (mm) and mean air temperature (°C) estimated from ERA5-Land for the collection site of *Hieronymiella bedelarii*, 2021–2024.

| Month | Precipitation (mm) | | | | Mean temperature (°C) | | | |
|---|---|---|---|---|---|---|---|---|
| | 2021 | 2022 | 2023 | 2024 | 2021 | 2022 | 2023 | 2024 |
| **Jan** | 246.93 | 172.5 | 57.43 | 52.45 | 15.12 | 15.78 | 15.94 | 17.15 |
| **Feb** | 152.27 | 165.72 | 75.41 | 124.07 | 14.94 | 14.8 | 14.83 | 16.14 |
| **Mar** | 149.56 | 213.25 | 74.57 | 246.32 | 14.21 | 14.62 | 14.93 | 16.17 |
| **Apr** | 129.94 | 64.05 | 18.25 | 81.68 | 13.76 | 14.66 | 14.86 | 16.54 |
| **May** | 12.89 | 15.81 | 17.25 | 17.3 | 13.74 | 12.89 | 15.17 | 15.95 |
| **Jun** | 17.61 | 7.42 | 1.18 | 1.19 | 12.44 | 13.25 | 13.42 | 15.44 |
| **Jul** | 10.66 | 6.08 | 5.69 | 9.9 | 12.57 | 15.28 | 14.7 | 13.21 |
| **Aug** | 14.04 | 19.79 | 9.9 | 5.84 | 14.14 | 14.35 | 15.77 | 15.26 |
| **Sep** | 51.99 | 23.74 | 8.92 | 33.14 | 16.59 | 15.04 | 18.09 | 17.26 |
| **Oct** | 55.68 | 26.73 | 14.13 | 85.91 | 17.11 | 16.02 | 19.25 | 18.07 |
| **Nov** | 143.05 | 5.74 | 36.49 | 129.24 | 16.20 | 16.63 | 19.21 | 16.92 |
| **Dec** | 256.52 | 53.84 | 103.42 | 147.41 | 14.89 | 15.92 | 17.31 | 16.15 |
| **Annual** | **1241.14** | **774.67** | **422.64** | **934.45** | **14.64** | **14.94** | **16.12** | **16.19** |

Data were extracted from the ERA5-Land grid cell nearest the collection locality (18.1° S, 65.7° W). Monthly precipitation values were converted from metres to millimetres, and temperature values from K to °C.

**Table S2**. Alkaloid profile of *H. bedelarii* characterized by UPLC–MS/MS.

| N° | Alkaloid | $[M+H]^+$ (m/z) | Major product ions (m/z) | RT (min) | %TIC |
|---|---|---|---|---|---|
| | ***Homolycorine-type*** | | | | |
| 1 | Candimine | 346 | 221, 243, 227 | 4.96 | 25.24 |
| | ***Lycorine-type*** | | | | |
| 2 | Lycorine | 288 | 147, 119, 177 | 2.30 | 8.19 |
| 3 | Galanthine | 318 | 162, 193, 237 | 6.56 | 13.48 |
| | ***Pretazettine-type*** | | | | |
| 4 | Pretazettine | 332 | 225, 300, 314 | 7.41 | 12.01 |
| 5 | Tazettine | 332 | 181, 314, 211, 282 | 10.45 | 10.16 |
| | ***Crinane/ haemanthamine-type*** | | | | |
| 6 | Haemanthamine | 302 | 226, 211, 196 | 7.65 | 8.80 |
| | ***Galanthamine-type*** | | | | |
| 7 | Galanthamine | 288 | 213, 198, 225 | 3.47 | 3.77 |
| | ***Not identified*** | | | | |
| 8 | NI (Homolycorine-type) | 316 | 298, 280, 239, 191 | 4.58 | 6.38 |
| 9 | NI | 376 | 256, 225, 239/241, 270/274, 316 | 9.11 | 3.24 |
| 10 | NI | 374 | 254, 272, 224, 211, 182 | 11.56 | 2.40 |

* Tentative. $ESI^+$, full-scan MS/MS; CE 30 eV. NI, not identified.

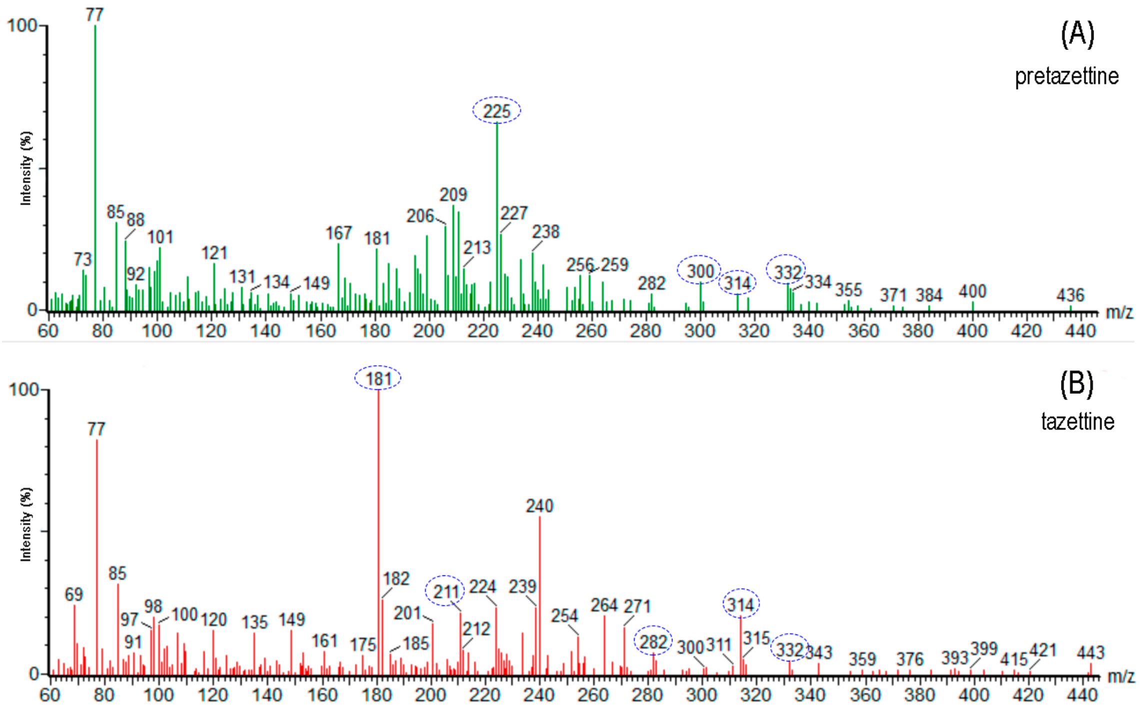


**Figure S1**. UPLC–MS/MS spectra of precursor ion *m/z* 332 $[M+H]^+$ corresponding to (A) pretazettine (RT = 7.41 min) and (B) tazettine (RT = 10.45 min).
